# Multidisciplinary Care after Acute Care for Stroke: A Prospective Comparison between a Multidisciplinary Post-Acute Care Group and a Standard Group Matched by Propensity Score

**DOI:** 10.3390/ijerph18147696

**Published:** 2021-07-20

**Authors:** Chong-Chi Chiu, Hsiu-Fen Lin, Ching-Huang Lin, Hong-Tai Chang, Hong-Hsi Hsien, Kuo-Wei Hung, Sheng-Li Tung, Hon-Yi Shi

**Affiliations:** 1 School of Medicine, College of Medicine, I-Shou University, Kaohsiung 82445, Taiwan; chiuchongchi@yahoo.com.tw; 2 Department of General Surgery, E-Da Cancer Hospital, Kaohsiung 82445, Taiwan; 3 Department of Neurology, Kaohsiung Medical University Hospital, Kaohsiung 80708, Taiwan; sflin@kmu.edu.tw; 4 Department of Neurology, Kaohsiung Medical University, Kaohsiung 80708, Taiwan; 5 Division of Neurology, Kaohsiung Veterans General Hospital, Kaohsiung 81341, Taiwan; chlin2524@vghks.gov.tw; 6 Department of Surgery, Kaohsiung Municipal United Hospital, Kaohsiung 80457, Taiwan; hongtchang@gmail.com; 7 Department of Business Management, National Sun Yat-sen University, Kaohsiung 80424, Taiwan; 8 Department of Internal Medicine, St. Joseph Hospital, Kaohsiung 80288, Taiwan; gavinhsien@gmail.com; 9 Division of Neurology, Department of Internal Medicine, Yuan’s General Hospital, Kaohsiung 80249, Taiwan; dimondwave@gmail.com; 10 Department of Medical Research, Chiayi Chang Gung Hospital, Chiayi 61301, Taiwan; vok4400@gmail.com; 11 Department of Healthcare Administration and Medical Informatics, Kaohsiung Medical University, Kaohsiung 80708, Taiwan; 12 Department of Medical Research, Kaohsiung Medical University Hospital, Kaohsiung 08708, Taiwan; 13 Department of Medical Research, China Medical University Hospital, China Medical University, Taichung 40402, Taiwan

**Keywords:** post-acute care, stroke, geriatric, functional status, risk factors

## Abstract

In this large-scale prospective cohort study, a propensity score matching method was applied in a natural experimental design to investigate how post-acute care (PAC) after stroke affects functional status and to identify predictors of functional status. The main objective of this study was to examine longitudinal changes in various measures of functional status in stroke patients and predictors of scores for these measures before and after PAC. A group of patients who had received PAC for stroke at one of two medical centers (PAC group, *n* = 273) was compared with a group who had received standard care for stroke at one of four hospitals (three regional hospital and one district hospital; non-PAC group, *n* = 273) in Taiwan from March, 2014, to October, 2018. The patients completed the functional status measures before rehabilitation, the 12th week and the 1st year after rehabilitation. Generalized estimating equations were used to estimate differences-in-differences models for examining the effects of PAC. The average age was 68.0 (SD = 8.1) years, and males accounted for 57.9%. During the follow-up period, significant risk factors for poor functional outcomes were advanced age, hemorrhagic stroke, and poor function scores before rehabilitation (*p* < 0.05). Between-group comparisons at subsequent time points revealed significantly higher functional status scores in the PAC group versus the non-PAC group (*p* < 0.001). Notably, for all functional status measures, between-group differences in total scores significantly increased over time from baseline to 1 year post-rehabilitation (*p* < 0.001). The contribution of this study is its further elucidation of the clinical implications and health policy implications of rehabilitative care after stroke. Specifically, it improves understanding of the effects of PAC in stroke patients at different follow-up times. Therefore, a policy implication of this study is that standard care for stroke should include intensive rehabilitative PAC to maximize recovery of overall function.

## 1. Introduction

Stroke causes a wide range of neurologic deficits and is the leading cause of functionality loss (disability and mobility) worldwide [1]. Stroke can be divided into two types: intracerebral hemorrhagic (ICH) stroke and ischemic (IS) stroke. The ICH type comprises 10–24% of all strokes and is associated with increased mortality [2,3,4]. Previous studies also show that patients with ICH type have a worse functional outcome compared to their IS counterparts, but the difference decreases over time and becomes non-significant after several years [3,4].

Post-acute care (PAC) plans have demonstrated effectiveness in helping patients return home after stroke and in improving and accelerating recovery of function [5]. In Taiwan, the National Health Insurance Administration implemented the Post-acute Care-Cerebrovascular Diseases (PAC-CVD) program in 2014 to improve allocation of medical resources to patients with these diseases and to improve outcomes. Apparently, however, no studies have compared the optimal duration and intensity of organized multidisciplinary neurological/rehabilitative care delivered by a regional/district hospital versus the standard rehabilitative care delivered by the neurology/rehabilitation ward of a medical center.

Although some studies have discussed positive contributions of PAC to stroke outcome, most studies have only used longitudinal data for two time points, and analyses of functional status predictors have been limited to only 1 year or less [6,7,8]. Another issue is that most studies have been published in regional rather than international journals. Finally, the samples investigated in related studies have been small, or limited to patients treated at a single institution [6,7,8]. 

To address the above limitations, this observational multicenter prospective cohort study addressed the following two questions:

1. Is a multidisciplinary PAC program delivered early after stroke effective in restoring function?
2. Should intensive rehabilitative PAC for stroke routinely include interventions for overall improvement of functional status?

## 2. Materials and Methods

### 2.1. The PAC Program

According to the PAC-CVD program guidelines, a patient receiving treatment at a medical center for the post-acute phase of stroke must be transferred to a regional hospital or a district hospital in which a PAC program had been established. The PAC therapy was delivered in a total of three sessions of complex therapeutic physical activities per day. Each PAC patient received 1 h of therapy (i.e., physical, occupational, or speech-swallowing therapy) per session. The specific rehabilitative components of the program were tailored to the individual patient, but all patients received facilitation, passive range of motion exercise, strengthening, and therapeutic exercise. Additionally, all participants underwent rehabilitation in a separate facility designed specifically for PAC delivery in patients with stroke. Thus, participating hospitals received function-related reimbursement packages to compensate for daily expenditures incurred for care of patients with stroke, including rehabilitation and management of associated comorbidities/complications. The PAC group and the non-PAC group completed the same rehabilitation program, but the non-PAC group received one session of therapy (physical, occupational, and speech/swallowing) a day, whereas the PAC group received three sessions of therapy a day. General hospitals have a financial motivation for transferring patients with stroke to lower-level community hospitals. However, transfers may be constrained by various factors, including the hospital policy for transferring PAC-CVD patients, the willingness of patients and their families to assume the burden of PAC, and the advice of the physician. Therefore, healthcare providers should understand how hospital stays and transfer policies are affected by these policies, particularly since a short length of stay (LOS) may not indicate a good outcome. The PAC group in this study received per-diem reimbursement, and the non-PAC group received fee-for-service reimbursement.

### 2.2. Sample and Study Design

**Figure 1:**
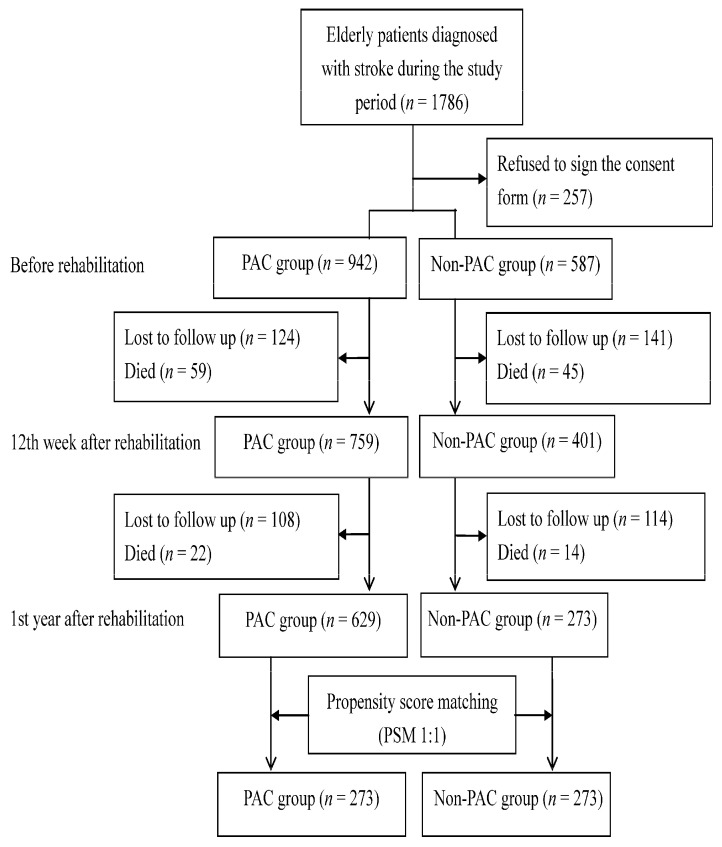
Flow chart showing population changes during the study, including patients who met initial exclusion criteria, those who later declined to participate and those who lost to follow-up.

The subjects of this study were a south Taiwan population of patients admitted to a PAC ward at one of two medical centers, to a non-PAC ward at one of three regional hospitals, or to district hospital within 30 days after stroke onset and during the period from March, 2014, to October, 2018. Additional inclusion criteria were an ICD-9-CM code for stroke (ischemic stroke codes 433.x, 434.x, or 436.x and hemorrhagic stroke codes 430 or 431), age 60 years or older, and a Modified Rankin Scale (MRS) score of 2–4. The MRS score ranges from 0 to 6, and a high MRS score indicates that severity of disability is high [9]. Patients were excluded if PAC beds were unavailable at the participating hospitals or if they had been transferred to PAC wards at other hospitals. Figure 1 shows that the final population of patients with a stroke diagnosis recruited for this study was 1786 patients. The institutional review board at all participating hospitals approved the study protocol. Additionally, all participants gave informed consent before they enrolled in this study. 

### 2.3. Instruments and Measurements

Functional disability is defined as the inability to perform certain daily life activities such as dressing, performing self-care activities, and ascending/descending stairs. In this study, Barthel Index (BI) score was used as a measure of functional disability [10]. The 10-item BI instrument has a maximum score of 10, which indicates complete independence. The Functional Oral Intake Scale (FOIS) was used to assess oral intake function in patients with dysphagia [11]. The FOIS is a 10-item scale that classifies swallowing function from 1 (nothing per oral) to 7 (unrestricted total oral intake). Cognitive status was quantified by the Mini-mental State Examination (MMSE) [12]. The MMSE is a measure of orientation, memory, attention, calculation, language, and construction functions. The total score ranges from 0 to 30; a high total score indicates good functional status. The Instrumental Activities of Daily Living Scale (IADL) indicates current function as well as functional improvement or deterioration over time [13]. The EuroQoL Quality of Life Scale (EQ-5D) is a scale for measuring self-assessed mobility, self-care, daily activities, pain or discomfort, and anxiety or depression as part of a total health state [14]. Each item is scored from 1 to 3 (not problematic, somewhat problematic, and highly problematic, respectively). The Berg Balance Scale (BBS) is a 14-item scale used to assess functional balance [15]. Items are scored from 0 (poor) to 4 (good), and the maximum score is 56. In each participant, all six instruments were administered before rehabilitation, 12 weeks after rehabilitation, and 1 year after rehabilitation. Notably, this study used the Chinese versions of the instruments, which are well-validated and are widely used in both clinical practice and research [5,7].

In all patients, functional status measures were collected before and after rehabilitation by their physiatrists and therapists. The covariates were age, gender, education, body mass index, stroke type, hypertension, hyperlipidemia, diabetes mellitus, atrial fibrillation, previous stroke, acute care LOS, LOS during rehabilitation, and pre-rehabilitation functional status of the patient. 

### 2.4. Statistical Analysis

The unit of analysis in this study was the individual patient with stroke. First, patients with stroke were characterized by tabulating descriptive statistics. For comparison, a group of PAC patients with stroke and a group of non-PAC patients with stroke were then selected by propensity score matching (PSM) [16]. In the current study, logistic regression modeling was used to calculate a propensity score for each patient with stroke. A 1:1 nearest-neighbor matching procedure was then used to match patients with stroke in the PAC group to similar patients with stroke in the non-PAC group according to the calculated propensity scores. Change trends over time in all functional status measures were summarized with box plots and descriptive statistics. 

A common limitation of longitudinal studies is the absence of an appropriate statistical methodology that can control for censoring and inter-correlations that occur when measures are repeatedly obtained for the same pool of subjects. To address this limitation, this study used a generalized estimating equations (GEE) model to cluster patients with stroke who had received treatment from the same physician. The GEE model was also useful for generating propensity scores that could be used to predict patients who had completed the PAC program. Additionally, total scores for each measure of functional status were compared between the PAC group and the non-PAC group by using differences-in-differences (DID) methodology (i.e., a pre-post study design with a comparison group). The GEE method also was used with a proper distribution [17]. The advantage of the GEE method is that it considers intra-class correlations when the same patients are repeatedly observed and when patients within the same matched pairs are repeatedly observed. Since functional status outcomes tend to be right-skewed, the GEE model used for data analysis in this study integrated a logarithmic link function and a gamma distribution. For a clear comparison of the two matched groups, values obtained in regression analyses were compared in the period from the day of stroke diagnosis until the end of the 1-year follow up. Standard errors in differences and standard errors in DID in the predicted values were estimated by bootstrap technique (1000 replications and sample sizes equal to the original sample size) [18]. The software package used to perform GEE in all statistical analyses was xtgee in Stata, version 13.0. All tests were two-sided, and *p* values less than 0.05 were considered statistically significant.

## 3. Results

In the patients who completed this 1-year study and in the patients lost to follow up during the period from 12 weeks to 1 year post-rehabilitation, patient characteristics and total scores for the above measures of functional status did not significantly change after completion of the rehabilitation program (data not shown). As Table 1 shows, some of the assessed characteristics revealed significant between-group differences before PSM (*p* < 0.05), but none revealed significant between-group differences after PSM. 

**Figure 2:**
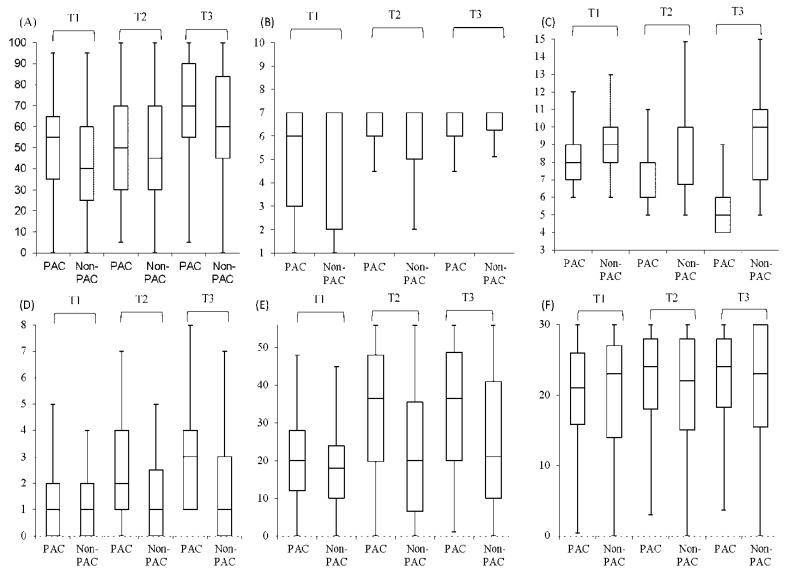
Comparison of box plot of all function status measures between post-acute care (PAC) group (*n* = 273) and non-PAC group (*n* = 273) before and after rehabilitation. The box plot indicates the interquartile range; the line, the median; the error bars, 95% confidence intervals (95% CIs). (**A**) BI, Barthel Index (**B**) FOIS, Functional Oral Intake Scale (**C**) EQ5D, EuroQoL Quality of Life Scale (**D**) IADL, Instrumental Activities of Daily Living Scale (**E**) BBS, Berg Balance Scale (**F**) MMSE, Mini-mental State Examination. T1: before rehabilitation; T2: 12th week after rehabilitation; T3: 1st year after rehabilitation.

Figure 2 compares total scores (with median and interquartile ranges) for functional status between the PAC group and non-PAC group during the period from before rehabilitation to 1 year post-rehabilitation. Notably, total scores obtained before rehabilitation for all functional status measures were similar in the two groups. From 12 weeks after rehabilitation to 1 year after rehabilitation, the PAC group had higher total scores for BI, FOIS, IADL, BBS, and MMSE compared to the non-PAC group. That is, the PAC group had larger improvements in all functional status measures except EQ-5D. According to the DID values (with mean and standard errors) for the PAC group and non-PAC group, the PAC program had significant (*p* < 0.001) positive net effects on BI, FOIS, IADL, BBS, and MMSE but had a significant (*p* < 0.001) negative net effect on EQ-5D during the study period (Table 2). Additionally, differences gradually decreased over time from −1.33 to −2.03. In the period form before rehabilitation until 1 year after rehabilitation, total scores for all functional status measures significantly (*p* < 0.001) differed between the two groups. All differences increased over time with the exception of EQ-5D. 

In Table 3, the multivariate analysis results are given for effective predictors of total scores for each functional status measure in the patients with stroke. After controlling for related variables, the PAC group had significantly higher scores for all functional status measures except EQ-5D in comparison with the non-PAC group (*p* < 0.05). The extent to which pain and loss of physical and social functions after rehabilitation interfered with activities of daily living had a significant positive association with age (*p* < 0.01).

Compared to hemorrhagic stroke, ischemic stroke was associated with significantly lower EQ-5D scores and significantly higher IADL scores (*p* < 0.05). Finally, pre-rehabilitation scores for each functional status measure were significantly and positively associated with post-rehabilitation scores for each functional status measure (*p* < 0.001).

## 4. Discussion

A current literature review indicates that this work is the first large cohort study to investigate the role of PAC in the functional status of patients with stroke by applying the PSM method in a natural experimental design [7,9]. During the follow-up period, risk factors for low functional status after rehabilitation included advanced age, hemorrhagic stroke, and low total score for each functional status measure before rehabilitation. After rehabilitation, the PAC group had superior functional status scores compared to the non-PAC group. Notably, the between-group difference in the total score for each functional status measure increased in the period from 12 weeks post-rehabilitation to 1 year post-rehabilitation.

This study makes three notable contributions to the growing literature on PAC. First, this study is one of the few to report longitudinal effects of the PAC program. Previous studies have only examined how PAC affects functional outcomes and were performed without comparison groups [4,5,6,7,8]. A novel feature of this study is its use of longitudinal data to compare the total scores for the measures of functional status between the PAC and non-PAC groups. Second, in analyses of follow-up data performed in other longitudinal studies, appropriate statistical methodologies are rarely applied to control for censoring and inter-correlations arising from repeated measures obtained from the same patient pool [17,18,19]. This study is the first to use a GEE model for examining longitudinal changes in the total score for each functional status measure and the first to explore how these changes are related to effective predictors of functional status after stroke. Finally, this prospective study analyzed longitudinal data for a relatively larger geriatric population of patients with stroke compared to previous works. The large sample size provided the statistical power needed to reveal long-term effects of a PAC program.

Throughout the study period, the PAC group had higher functional status scores compared to the non-PAC group. Other studies have also reported that PAC improves stroke outcomes [5,7]. For example, Miyai et al. (2011) reported that an intensive PAC plan integrated in the Japanese medical insurance system after 2000 improved stroke rehabilitation outcomes and that improved as the duration of PAC improved [20]. The Taiwan NHI system currently limits patients with stroke to a once-daily session of therapy (physical, occupational, or speech). For the PAC group analyzed in our study, however, the only limitation on therapy was the tolerance of the individual patient. As a result, the patients who had the highest tolerance for intensive rehabilitation after stroke achieved the most rapid restoration of function and had the fewest complications. 

In the United States, post-acute inpatient rehabilitation facility care, which is 3 h/day, obtains better functional outcomes than post-acute skilled nursing facility care, which is 60–90 min/day this guideline is similar with our PAC-CVD program. Accordingly, patients in our PAC-CVD program obtained better functional outcomes compared to patients who had received PAC in skilled nursing facilities [21]. In comparison with patients treated for stroke at a general ward, patients treated at a stroke unit reportedly have a shorter hospital stay and had lower mortality for as long as 1 year after stroke [22]. Moreover, colocation of acute care and rehabilitative care for stroke in a single district hospital was associated with reduced mortality and reduced LOS [23]. In Taiwan, however, colocation of acute and rehabilitative care in a single medical center may be more costly than intensive PAC in regional or district hospitals. 

Age was an independent predictor of outcomes in the PAC group. Our data were consistent with reports that improvements in health outcomes obtained by rehabilitation tend to decrease as age increases [7,24]. Notably, patients tended to have less social support and more co-morbidities, and number of co-morbidities is a controlled variable in GEE models. Selection bias may be a contributing factor in the observed improvements in health outcomes. Notably, most of the patients with stroke investigated in this study were males who were aged 65 to 75 years and who had risk factors for stroke, e.g., hypertension, diabetes, or obesity. All of these patient characteristics are statistically associated with quality of life (*p* < 0.05). According to Ministry of Health and Welfare, 79% to 86% of patients with stroke show an improvement in quality of life [7]. The average score of the BI improved from 39.1 to 63.7. In the current study, comparisons of the PAC and non-PAC groups at different follow-up time points showed that both groups improved after admission, but the improvement was larger in the PAC group. 

Patients with ischemic stroke had larger functional status improvements compared to those with hemorrhagic stroke, which is compatible with reports of a strong negative association between hemorrhagic stroke and functional status [25,26]. Thus, compared to ischemic stroke, hemorrhagic stroke is usually more severe at the time of onset and has a higher mortality rate.

During follow up, pre-rehabilitation functional status was the best predictor of total scores for all functional status measures during follow up, which is consistent with earlier works [5,7,27]. That is, independence of function after stroke requires recovery from impairment in all functional domains. Therefore, to improve functional outcomes during the chronic phase after stroke, rehabilitation should be designed to assess and reduce impairment in all functional domains simultaneously. The observed importance of simultaneous recovery in multiple functional domains also suggests that rehabilitative programs for improving activities of daily living and walking can substantially improve functional status in all patients with stroke. Early rehabilitation is essential in patients with stroke for two reasons. First, motor dysfunction resulting from stroke limits physical activity. Over time, mobility is reduced by muscle atrophy, joint stiffness, limb shrinkage, and other sequelae. Second, despite reports that most improvements occur within 6 months after stroke, the patients who received standard care for 6 months had significant improvements in functional status outcomes [7,28]. Therefore, researchers surmise that therapy beyond 6 months after stroke can still improve recovery.

The strength of this study is the use of a large multicenter geriatric population to investigate functional status after an initial stroke. Given the large population size in this study, the findings are likely applicable in populations with similar characteristics in countries elsewhere. In-person interviews with all participants enabled accurate collection of data and accurate evaluation of functional outcomes. The sample investigated in this study comprised patients who had received acute care for 30 days after stroke onset. All patients in this study were recruited from four hospitals (one regional hospital and one district hospital) in Taiwan, and the four hospitals were those with the largest populations of patients under acute care for stroke. Analysis of a sample selected from four different institutions ensured that patient outcomes would not be dependent on physician experience and skill. Furthermore, although this study revealed that intensive strength training was associated with good functional recovery in both PAC and non-PAC patients, cluster-randomized trials are needed for more rigorous analyses of the effects of rehabilitation intensity on stroke recovery, particularly given the relatively high cost effectiveness of rehabilitative PAC administered under per-diem reimbursement. Despite the limitations noted above, this study can be considered an important contribution to the literature on factors in stroke outcome, and it further expands the evidence base required for further comparisons of care and outcomes in geriatric populations of patients with stroke in different countries. 

## 5. Conclusions and Implications 

Although the incidence of stroke is high, PAC provided early after stroke can improve restoration of function. In addition to improving health, early rehabilitation can improve confidence and self-care ability in these patients. One health policy implication of this study is that routine treatment for stroke should include intensive rehabilitative PAC and that PAC should focus on improving overall functional status. Overall, the PAC group revealed significantly larger improvements in mean scores for functional outcome measures compared to the non-PAC group, which is an important contribution to the literature and a novel initial finding that will hopefully inspire further research to clarify the role of PAC. The results of this study have practical applications in establishing comprehensive and systematic programs for care if patients with stroke. However, the cost effectiveness of a PAC program for stroke relative to non-PAC programs needs further study. 

## Figures and Tables

**Table 1:** Stroke patient characteristics.

		Before Propensity Score Matching			After Propensity Score Matching		
Variables		PAC Group (*n* = 942)	Non-PAC Group (*n* = 587)	*p* Value	PAC Group (*n* = 273)	Non-PAC Group (*n* = 273)	*p* Value
Age, years		69.3 ± 8.5	68.8 ± 7.6	0.736	69.1 ± 8.3	68.9 ± 8.0	0.834
Gender	Female	403 (42.8%)	238 (40.6%)	0.704	116 (42.4%)	114 (41.9%)	0.684
	Male	539 (57.2%)	349 (59.4%)		157 (57.6%)	159 (58.1%)	
Education, years		7.8 ± 4.3	9.4 ± 4.5	<0.001	8.9 ± 4.1	9.2 ± 4.4	0.888
BMI, kg/m^2^		24.4 ± 3.2	24.4 ± 3.9	0.976	24.0 ± 3.0	23.9 ± 3.3	0.849
Stroke type	Ischemic	806 (85.6%)	512 (87.2%)	0.669	232(85.0%)	236 (86.4%)	0.891
	Hemorrhagic	136 (14.4%)	75 (12.8%)		41 (15.0%)	37 (13.6%)	
Hypertension	Yes	677 (71.9%)	421 (71.8%)	0.995	196 (72.0%)	196 (72.0%)	0.997
Hyperlipidemia	Yes	265 (28.1%)	273 (46.5%)	<0.001	114 (41.8%)	117 (42.8%)	0.507
Diabetes mellitus	Yes	343 (36.4%)	338 (57.6%)	0.270	103 (37.7%)	102 (37.4%)	0.990
Atrial fibrillation	Yes	78 (8.3%)	49 (8.3%)	0.989	22(8.0%)	17 (6.3%)	0.534
Previous stroke	Yes	131 (13.9%)	146 (24.9%)	<0.001	49 (18.0%)	50 (18.4%)	0.879
Acute care LOS, days		13.01 ± 27.83	24.45 ± 34.61	<0.001	23.75 ± 11.84	24.50 ± 11.56	0.356
LOS during rehabilitation, days		31.52 ± 17.75	37.1 ± 12.59	<0.001	35.52 ± 12.04	36.63 ± 11.91	0.916
BI score before rehabilitation		41.91 ± 23.10	34.67 ± 23.48	<0.001	34.90 ± 20.00	34.43 ± 17.80	0.879
FOIS score before rehabilitation		5.95 ± 3.04	5.38 ± 2.25	<0.001	5.57 ± 2.80	5.13 ± 2.75	0.974
EQ5D score before rehabilitation		10.67 ± 1.86	10.40 ±1.78	0.015	10.81 ± 1.90	10.87 ± 2.15	0.891
IADL score before rehabilitation		1.41 ± 1.20	1.15 ± 1.12	<0.001	1.32 ± 1.14	1.13 ± 1.01	0.934
BBS score before rehabilitation		15.30 ± 14.99	16.91 ± 17.27	0.097	16.13 ± 14.08	16.67 ± 15.57	0.882
MMSE score before rehabilitation		20.15 ± 7.90	18.50 ± 9.66	0.001	20.67 ± 11.50	19.57 ± 10.20	0.859

Data presented as mean ± standard deviation (SD) or n (%). Abbreviations: BMI = body mass index; LOS = length of stay; PAC = post-acute care; BI = Barthel Index; FOIS = Functional Oral Intake Scale; EQ5D = EuroQoL Quality of Life Scale; IADL = Instrumental Activities of Daily Living Scale; BBS = Berg Balance Scale; MMSE = Mini-mental State Examination. *p* values are calculated using the independent *t* test or X^2^ test.

**Table 2:** Comparison of functional status between PAC group (*n* = 273) and non-PAC group (*n* = 273) before and after rehabilitation.

Functional Status Measures		Before Rehabilitation	After Rehabilitation		12th Week after Rehabilitation before Rehabilitation		1st Year after Rehabilitation before Rehabilitation	
			12th Week	1st Year				
		Mean	Mean	Mean	Mean	Standard Error	Mean	Standard Error
BI	PAC	51.90	59.32	65.76	7.42	1.95	13.86	4.84
	Non-PAC	50.79	54.20	60.29	3.41	1.18	9.50	3.66
	Difference	1.12	3.12	3.47	4.01	0.88	4.36	0.91
FOIS	PAC	5.38	6.39	6.52	1.01	0.12	1.14	0.19
	Non-PAC	5.19	5.74	5.95	0.55	0.11	0.76	0.15
	Difference	0.19	0.65	0.58	0.46	0.10	0.38	0.07
EQ-5D	PAC	8.41	6.28	4.99	−2.13	0.43	−3.42	0.58
	Non-PAC	8.92	8.12	7.53	−0.80	0.42	−1.39	0.41
	Difference	−0.50	−1.84	−2.54	−1.33	0.16	−2.03	−0.26
IADL	PAC	1.58	2.85	3.42	1.27	0.11	1.84	0.12
	Non-PAC	1.17	2.36	2.90	1.19	0.18	1.73	0.20
	Difference	0.40	0.50	0.52	0.08	0.03	0.11	0.03
BBS	PAC	20.59	29.53	34.40	8.94	1.32	13.81	4.25
	Non-PAC	18.89	24.85	29.60	5.96	1.64	10.71	2.38
	Difference	1.70	4.68	4.80	2.98	0.68	3.10	0.83
MMSE	PAC	20.72	22.46	23.13	1.74	0.20	2.41	0.22
	Non-PAC	19.41	20.77	21.15	1.36	0.18	1.74	0.13
	Difference	1.31	1.69	1.98	0.38	0.10	0.67	0.10

Abbreviations: PAC = post-acute care; BI = Barthel Index; FOIS = Functional Oral Intake Scale; EQ5D = EuroQoL Quality of Life Scale; IADL = Instrumental Activities of Daily Living Scale; BBS = Berg Balance Scale; MMSE = Mini-mental State Examination. Generalized estimating equation (GEE) models with gamma distribution were used to predict values. All *p* values are <0.001.

**Table 3:** Coefficients of GEE models of effective predictors of each functional status measure in patients with stroke over a 1-year period after propensity score matching (*n* = 546).

Variables	BI	FOIS	EQ-5D	IADL	BBS	MMSE
Group PAC vs. non-PAC	2.24 *	0.46 *	−0.47 **	0.15 **	1.94 *	0.27 *
Gender Male vs. female	0.01	0.02	−0.02	0.15	0.76	0.07
Age, years	−0.20 **	−0.02	0.02 ***	−0.01 **	−0.17 ***	−0.07 **
Education, years	0.12	0.01	0.04	0.01	−0.13	0.02
BMI, kg/m^2^	0.23	0.01	0.01	0.02	0.07	0.07
Stroke type Ischemic vs. hemorrhagic	0.14	0.15	−0.43 *	0.23 *	−0.08	−0.18
CCI, score	1.78	−0.01	−0.03	−0.19	1.42	−0.33
Length of stay, days	0.04	−0.01	0.01	−0.01	0.02	0.01
UTI Yes vs. no	−1.56	−0.04	0.30	0.02	−0.86	1.11
Renal disease Yes vs. no	0.41	0.06	0.18	0.01	−1.27	0.24
Hypertension Yes vs. no	−2.26	0.03	−0.05	0.04	−1.67	0.07
Diabetes Yes vs. no	−0.18	0.05	0.14	0.23	−0.33	0.64
Hyperlipidemia Yes vs. no	1.80	0.13	−0.11	0.09	0.29	−0.06
Functional status before rehabilitation	0.82 ***	0.69 ***	−0.74 ***	0.86 ***	0.83 ***	0.73 ***

Abbreviations: GEE = generalized estimating equations; BI = Barthel Index; FOIS = Functional Oral Intake Scale; EQ-5D = EuroQoL Quality of Life Scale; IADL = Instrumental Activities of Daily Living Scale; BBS = Berg Balance Scale; MMSE = Mini-Mental State Examination; PAC = post-acute care; BMI = body mass index; CCI = Charlson comorbidity index; UTI = urinary tract infection. * *p* < 0.05, ** *p* < 0.01, *** *p* < 0.001.

## Data Availability

The data presented in this study are available on request from the corresponding author.
